# Sphingosine-1-phosphate signaling mediates shedding of measles virus-infected respiratory epithelial cells

**DOI:** 10.1128/jvi.01880-24

**Published:** 2025-03-27

**Authors:** Jacqueline K. Brockhurst, Brittany E. Salciccioli, Diane E. Griffin

**Affiliations:** 1 Department of Molecular and Comparative Pathobiology, Johns Hopkins School of Medicine 1500 , Baltimore, Maryland, USA; 2 Department of Molecular Microbiology and Immunology, Johns Hopkins Bloomberg School of Public Health 25802 , Baltimore, Maryland, USA; University of Kentucky College of Medicine, Lexington, Kentucky, USA

**Keywords:** measles, respiratory epithelial cells

## Abstract

Measles virus (MeV) is an extremely infectious respiratory virus and a major cause of childhood morbidity and mortality worldwide. MeV infection of the respiratory epithelium induces shedding of multinucleate epithelial cells from the apical surface of the epithelium without compromising epithelial barrier integrity. To study the mechanisms driving the apical extrusion of MeV-infected respiratory epithelial cells, we used primary differentiated tracheal epithelial cell cultures (rhTECs) and respiratory samples from rhesus macaques infected with wild-type MeV (WT MeV) or live-attenuated MeV (LAMV). We show that sphingosine-1-phosphate (S1P) signaling, rather than cell death or inflammasome activation, plays a key role in WT MeV and LAMV-induced cell shedding. Inhibiting S1P signaling resulted in delayed shedding of clusters of infected cells and higher viral titers within the epithelium, suggesting that cell extrusion impacts viral dynamics within the respiratory tract. We also found that shedding of individual infected cells began early after apical infection, prior to the formation of infected cell clusters within the epithelium. These findings offer new insights into MeV biology and pathogenesis within the respiratory tract.

**IMPORTANCE:**

Despite the availability of a safe and effective vaccine, measles virus (MeV) still has a significant global impact, and in 2022 alone led to over 136,000 deaths. MeV is one of the most contagious known viruses and spreads via the respiratory route. When respiratory epithelial cells are infected, they are shed into the lumen of the respiratory tract, but this process is poorly understood. Here, we use primary differentiated respiratory epithelial cells from rhesus macaques to show that sphingosine-1-phosphate (S1P) signaling, and not cell death or inflammasome activation, plays a role in cell shedding during both wild-type and live-attenuated MeV infection. Through this mechanism, MeV-infected cells are extruded without disrupting the integrity of the respiratory epithelium. Inhibiting S1P signaling resulted in delayed shedding of infected cells and higher viral titers in the epithelium. These findings indicate that host cellular responses play an important role in MeV infectivity.

## INTRODUCTION

Measles, the disease caused by measles virus (MeV), a single-stranded, negative-sense RNA virus in the family *Paramyxoviridae*, is a highly transmissible respiratory infection. MeV is spread by droplets and aerosols, with infectious virus capable of remaining airborne for up to 2 hours (1–3). A single infected person can transmit virus to an estimated 9–18 susceptible individuals, making MeV one of the most infectious viruses known to man (4, 5). Despite the availability of an effective vaccine, disruption to surveillance and immunization programs during the COVID-19 pandemic and rising vaccine hesitancy have led to the lowest worldwide first-dose measles vaccine coverage levels since 2008 (5, 6).

After entering the respiratory tract, wild-type (WT) MeV uses CD150 (SLAM) receptors (7), expressed on alveolar macrophages, dendritic cells, and lymphocytes, or nectin-4 (8, 9) on the basolateral surface of epithelial cells to invade host cells. Studies in primary differentiated airway epithelial cell (AEC) cultures have demonstrated that MeV infection from either the apical or basolateral surface of the polarized respiratory epithelium induces shedding of infected multinucleate cells from the apical surface of the cultures (10, 11). Despite the loss of cells, the integrity of the epithelium remains intact throughout infection. In addition, shed cells remain viable even 48 h after leaving the adherent epithelium (10, 11). The presence of multinucleate epithelial cells in the respiratory tract has long been noted during MeV infection in children; cells can be detected in nasopharyngeal and other mucosal sites prior to development of the classic measles exanthema (12, 13).

The mechanism behind this MeV infection-induced apical shedding of epithelial cells is not well understood and may involve a number of different processes. The integrity of a healthy epithelium depends on barrier function maintenance despite constant cell turnover through loss and regeneration (14). During normal homeostatic turnover, apoptotic or overcrowded cells are extruded from the apical surface of the epithelium through a pathway mediated by sphingosine-1-phosphate (S1P) signaling (15–17). An apoptotic or mechanical stimulus within a cell stimulates activation of sphingosine kinase (SphK) production and release of the bioactive lipid S1P, which binds to S1P-receptor 2 (S1PR2) on neighboring cells. S1PR2, a G-protein coupled receptor, signals to induce the formation of an actin/myosin ring at the basolateral intercellular junctions surrounding the extruding cell, which contracts to squeeze the cell toward the apical surface of the epithelium. Simultaneously, the neighboring cells begin to reform intercellular junctions, maintaining epithelium barrier function despite loss of the extruded cell (15–17). Pathogens such as *Listeria monocytogenes* take advantage of homeostatic epithelial cell turnover to gain access to tight junctional proteins normally inaccessible from the apical surface (18). MeV may use a similar strategy to interact with the basolaterally expressed receptor nectin-4 from the apical surface of the respiratory epithelium.

Pathogen infection of epithelial cells may also induce cell shedding through non-homeostatic pathways. Uropathogenic *Escherichia coli* (UPEC) infection induces exfoliation of bladder epithelium through a cytolytic mechanism suggested to be an innate host response (19–21). Intestinal epithelial cells infected with *Salmonella typhimurium* are expelled following activation of the NAIP/NLRC4 inflammasome (22, 23). The apical shedding of respiratory epithelial cells has also been demonstrated in other viral infections, including respiratory syncytial virus (RSV) (24, 25) and SARS-CoV-2 (26), although the exact mechanisms by which these viruses trigger cell shedding are unknown.

In this study, we investigated the role of S1P signaling and pathogen-mediated exfoliation in MeV infection-induced apical shedding of respiratory epithelial cells. We used primary differentiated rhesus macaque tracheal epithelial cell (rhTEC) cultures and samples from macaques intratracheally inoculated with live-attenuated measles vaccine virus (LAMV) or WT MeV to model MeV infection in the airways and explore the mechanisms driving cell shedding. Given that LAMV replicates as efficiently as WT MeV in the respiratory epithelium and also induces cell shedding (10), we analyzed both LAMV and WT MeV infection of rhTECs. Our findings indicate that S1P signaling, rather than cell death or inflammasome activation, plays an important role in MeV-infection-induced apical cell shedding.

## RESULTS

### Cell death does not precede shedding of MeV-infected epithelial cells

**Fig 1:**
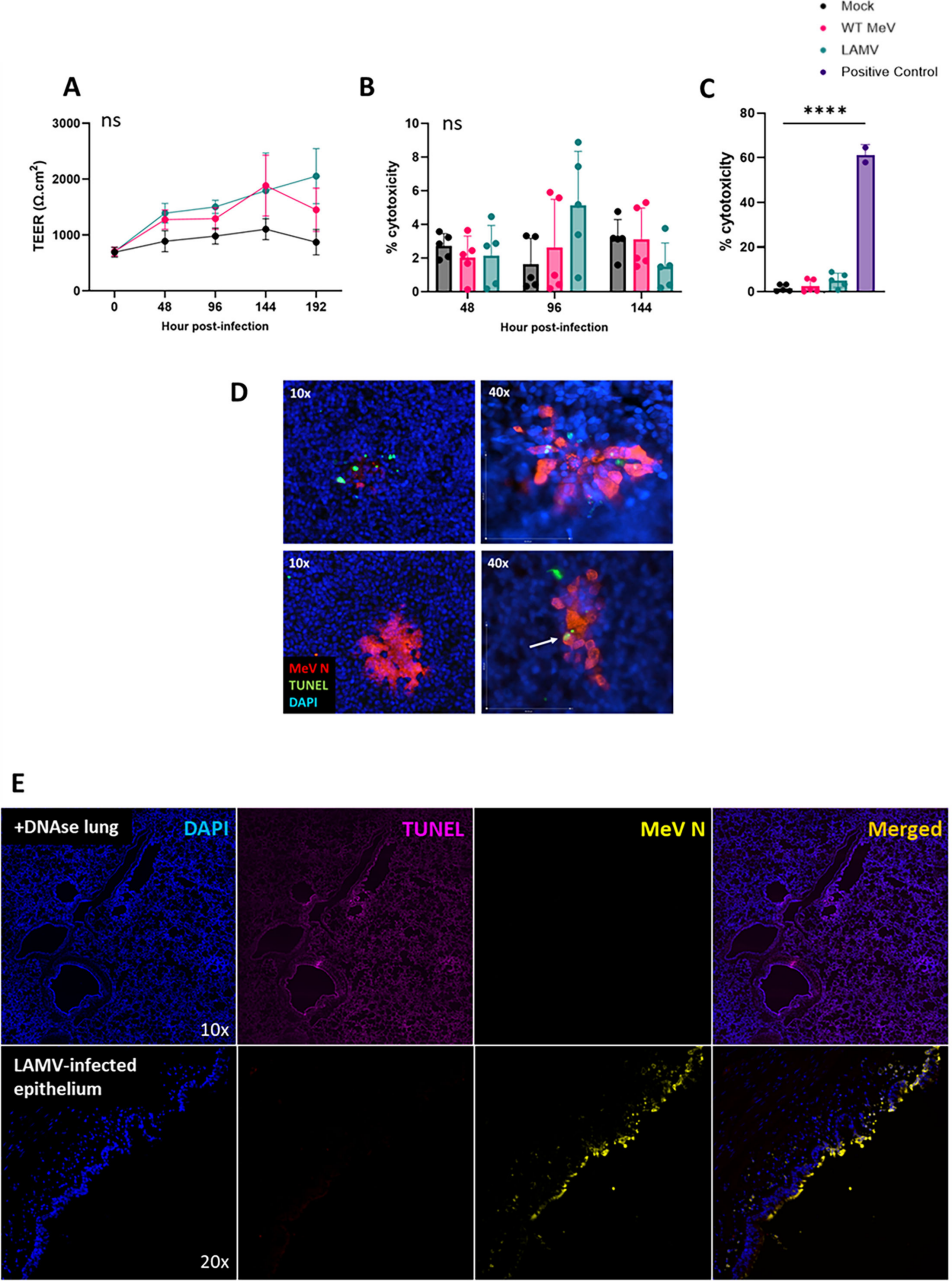
Cell death does not precede shedding of MeV-infected epithelial cells. rhTECs were apically infected with WT MeV or LAMV at an MOI of 1, and cytotoxicity was assessed. (A) Transepithelial electrical resistance (TEER) was measured throughout 144 h of infection. (B) LDH release by infected rhTECs was quantified over 144 hpi and (C) LDH release in infected cultures was compared to positive control cultures incubated with Triton-X 100 (*P* < 0.0001). (D) Immunofluorescent TUNEL staining was performed on WT- and LAMV-infected rhTEC epithelium, with representative images from WT-infected cultures shown here. TUNEL uptake by infected cells was infrequent. Occasionally, cells in the processes of extruding from the epithelium were TUNEL positive (white arrow). Images were obtained with a Zeiss AxioImager M2 fluorescent microscope. (E) Tracheal tissue from a macaque inoculated with LAMV was assessed for TUNEL uptake and MeV N protein. DNAse-treated lung tissue was used as a positive control for TUNEL stain. Images were obtained with a Leica Thunder Imager Live Cell fluorescent microscope. For rhTEC experiments, *n* = 2–3 replicate wells from two different donors. Significance was measured by two-way ANOVA with Tukey’s multiple comparisons.

While gross cytopathic effects are not observed in MeV-infected AEC epithelium (10, 11, 27), live-cell exfoliation driven by apoptosis-like signals or inflammasome activation has been demonstrated in other epithelial infections (19–23). To determine whether MeV-induced cell death signals trigger cellular exfoliation, we performed several assays to assess cell death pathways in the epithelium. As previously reported, transepithelial electrical resistance—a measure of epithelial barrier integrity—was maintained or even improved in rhTEC cultures throughout a 2 week infection with WT MeV and LAMV (Fig. 1A) (10, 27). Lactate dehydrogenase (LDH) release into the basolateral compartment of infected cultures was measured to evaluate cytotoxicity (28). Positive control samples for LDH release were generated by incubating rhTECs with Triton-X 100 for 24 h. There was no significant increase in LDH levels over the course of infection with either WT MeV or LAMV compared with mock-infected cultures (Fig. 1B), and LDH levels in infected cultures were lower than positive control samples (Fig. 1C, mean % cytotoxicity in positive control = 61%). These data confirm that there is no evidence of loss of epithelial integrity through disrupted cell-cell contact or plasma membrane integrity during either WT MeV or LAMV infection.

Because cell death signals trigger bladder epithelial cell exfoliation in UPEC infection (20, 21), we assessed DNA fragmentation with terminal deoxynucleotidyl transferase dUTP nick end labeling (TUNEL) in infected respiratory epithelium. In rhTEC cultures, TUNEL and MeV N proteins were not consistently co-localized; often infected cells had no evidence of TUNEL staining or only patchy TUNEL positivity (Fig. 1D). These results were consistent for both WT MeV and LAMV infections. The occasional TUNEL-positive cells were frequently detected in the plane above the epithelium, making it difficult to determine if nuclear fragmentation preceded cell shedding or occurred as a result of the cell losing contact with the epithelium (Fig. 1D, white arrow). We also examined formalin-fixed, paraffin-embedded (FFPE) tracheal tissues from macaques intratracheally inoculated with LAMV. No evidence of TUNEL staining was detected in foci of LAMV-infected cells within the respiratory epithelium (Fig. 1E), consistent with the rhTEC culture results.

### Inflammasome activation does not occur in MeV-infected respiratory epithelial cells

**Fig 2:**
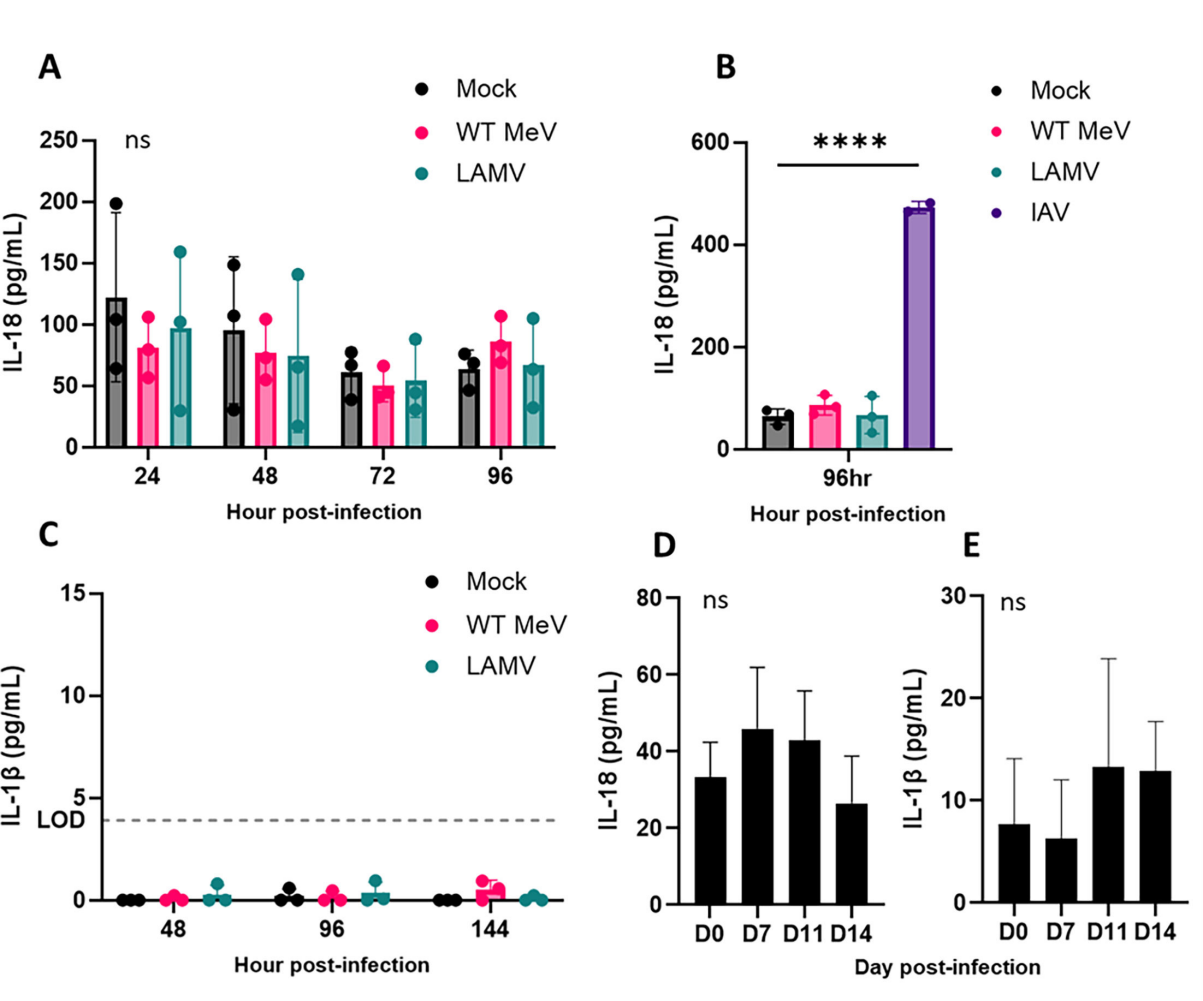
IL-18 and IL-1β production does not occur following MeV infection of the respiratory epithelium. Inflammasome activation products IL-18 and IL-1β were measured by ELISA in apical washes from WT- and LAMV-infected rhTECs (MOI 1; *n* = 3 replicate wells) (A, C) and BAL samples from WT-infected macaques (*n* = 4) (D, E). (B) IL-18 levels from rhTECs at 96 hpi were compared with influenza A virus (IAV)-infected rhTECs as a positive control (*P* < 0.0001). LOD = limit of detection. Significance was determined by two-way ANOVA (A, B) or one-way ANOVA (C, D, E) with Tukey’s multiple comparisons.

Inflammasome activation plays a role in intestinal epithelial cell shedding following infection with *Salmonella typhimurium* (22, 23). MeV induces NLRP3 inflammasome activation in primary human monocyte-derived macrophages and THP cells (29, 30). Both WT MeV- and LAMV-infected macrophages produced inflammasome products interleukin (IL)−18 and IL-1β without evidence of pyroptosis (30). We, therefore, examined the media of apically infected rhTECs for IL-18 and IL-1β as evidence of inflammasome activation. A strain of influenza A virus (IAV) known to induce inflammasome activation in AECs was used as a positive control. IL-18 and IL-1β levels produced by WT MeV- or LAMV-infected cultures were similar to mock-infected cultures (Fig. 2A and B) and IL-18 levels were low compared with IAV-infected cultures (Fig. 2C), suggesting that inflammasome activation does not occur as a response to WT MeV or LAMV infection of respiratory epithelial cells. Similarly, there was no increase in IL-18 or IL-1β (Fig. 2D and E) in bronchoalveolar lavage (BAL) fluid from the lungs of macaques infected with WT MeV. These results suggest that exfoliation of MeV-infected cells due to pathogen-induced cell death and subsequent loss of cell-cell/cell-matrix adhesions is not the major driving force of cell shedding during MeV infection.

### Inhibiting S1P signaling increases infectious MeV in the epithelium

**Fig 3:**
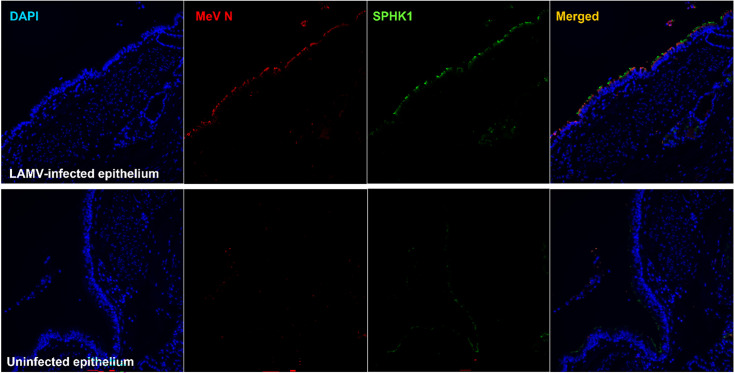
Sphingosine kinase was detected at higher levels in LAMV-infected epithelial cells in macaque tracheal tissue samples than in uninfected sections. FFPE tracheal sections were immunostained for MeV N protein and SPHK1 protein. Respiratory epithelial cells that stained positive for MeV N also stained positive for SPHK1, while little SPHK1 was detected in uninfected sections of epithelium.

**Fig 4:**
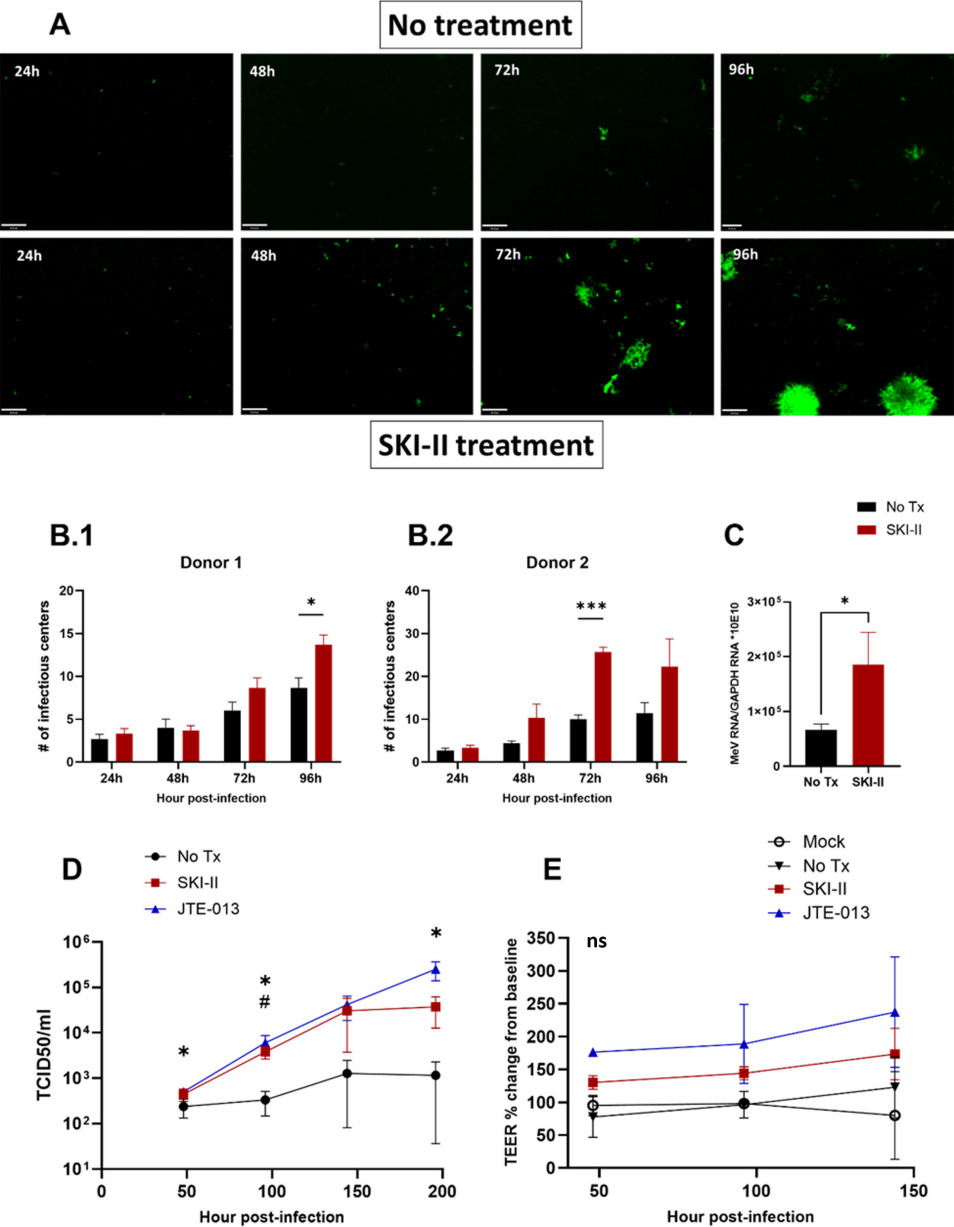
S1P signaling inhibition increased MeV in the epithelium. (A) rhTECs were apically infected with rGFP-LAMV, and SKI-II or vehicle was added to the basolateral media (MOI 1; *n* = 3 replicate wells per timepoint, from two donors). Representative images of infectious centers in the epithelium were obtained with a Zeiss AxioImager M2 fluorescent microscope. (B) The number of infectious centers per rhTEC was quantified in two donors (B.1 and B.2) (*P = 0.024; ***P = 0.0003). (C) MeV N RT-qPCR was used to quantify WT-MeV present in the epithelium of SKI-II-treated wells compared to untreated wells at 96 hpi (*P* = 0.027) (MOI 1; *n* = 3 replicate wells). (D) rhTECs were apically infected with WT MeV (MOI 1; *n* = 4 replicate wells per timepoint). MeV titers in inhibitor-treated and untreated epithelium were determined by co-cultivation of infected rhTECs on Vero-hSLAM cells (*JTE vs No Tx: *P* < 0.05; #SKI-II vs No Tx: *P* < 0.05). (E) TEER was measured in infected, S1P-signal-inhibited cultures over the course of infection. Significance was determined by two-way ANOVA (B, D, E) with Tukey’s multiple comparisons and unpaired t test (C). One-way ANOVA was used to determine significant changes in titers over time compared to baseline (D).

We next investigated the role of S1P-mediated epithelial cell extrusion in MeV-infected cell shedding. Expression of both SphK and S1P is transiently increased during infection of B cell lines by WT MeV (31, 32). We first immunostained LAMV-infected macaque tracheal epithelial tissue for both MeV N and SphK and found that SphK staining co-localized with foci of infection within the respiratory epithelium (Fig. 3). Little expression of SphK was detected in sections of uninfected epithelium. We then employed the SphK inhibitor SKI-II to disrupt S1P synthesis and the S1PR2 antagonist JTE-013 to inhibit S1P signaling. Because both LAMV and WT MeV replicate within the respiratory epithelium and induce cell-shedding (10), we hypothesized that S1P signaling inhibition would have similar results in both infection conditions. First, SKI-II was added to the basolateral media of infected rhTEC cultures after apical infection with rGFP-LAMV or WT MeV, and the amount of virus in the adherent epithelium was measured. Over the course of 96 h, rGFP-LAMV-infected cultures treated with SKI-II developed more clusters of infected cells in the epithelium than untreated cultures (Fig. 4A and Fig. 4B.1/B.2). Similarly, when MeV N RNA levels were examined at 96 hpi, SKI-II-treated cultures infected with WT MeV had higher RNA levels than infected, untreated cultures (Fig. 4C). These results suggested that SKI-II treatment inhibited shedding and increased retention of both LAMV and WT MeV-infected cells in the epithelium.

We then compared levels of replicating WT MeV present in the epithelium by co-cultivating trypsinized rhTECs with Vero cells stably expressing human MeV receptor CD150 (SLAM). Both SKI-II and JTE-013 treatments increased accumulation of infected cells in the epithelium compared with untreated cultures. Both JTE-013- and SKI-II-treated cultures accumulated higher levels of virus compared with untreated cultures (Fig. 4D). Furthermore, titers in infected, untreated cultures remained stable over the course of infection, while both SKI-II and JTE-013 treatment led to a significant increase in viral titers over time (one-way ANOVA: SKI-II: mean titer at 48 h vs 96 h = 444.3 vs 3814, *P* = 0.02; JTE: mean titer at 48 h vs 192 h = 503 vs 253216, *P* = 0.05). Despite the increased amount of virus present in the epithelium with S1P signal inhibition, TEER in these cultures was not significantly different from either mock or untreated cultures (Fig. 4E). These results suggest that S1P signaling is important for the removal of MeV-infected cells from the respiratory epithelium.

### Inhibition of S1P signaling decreases shedding of MeV-infected cells

**Fig 5:**
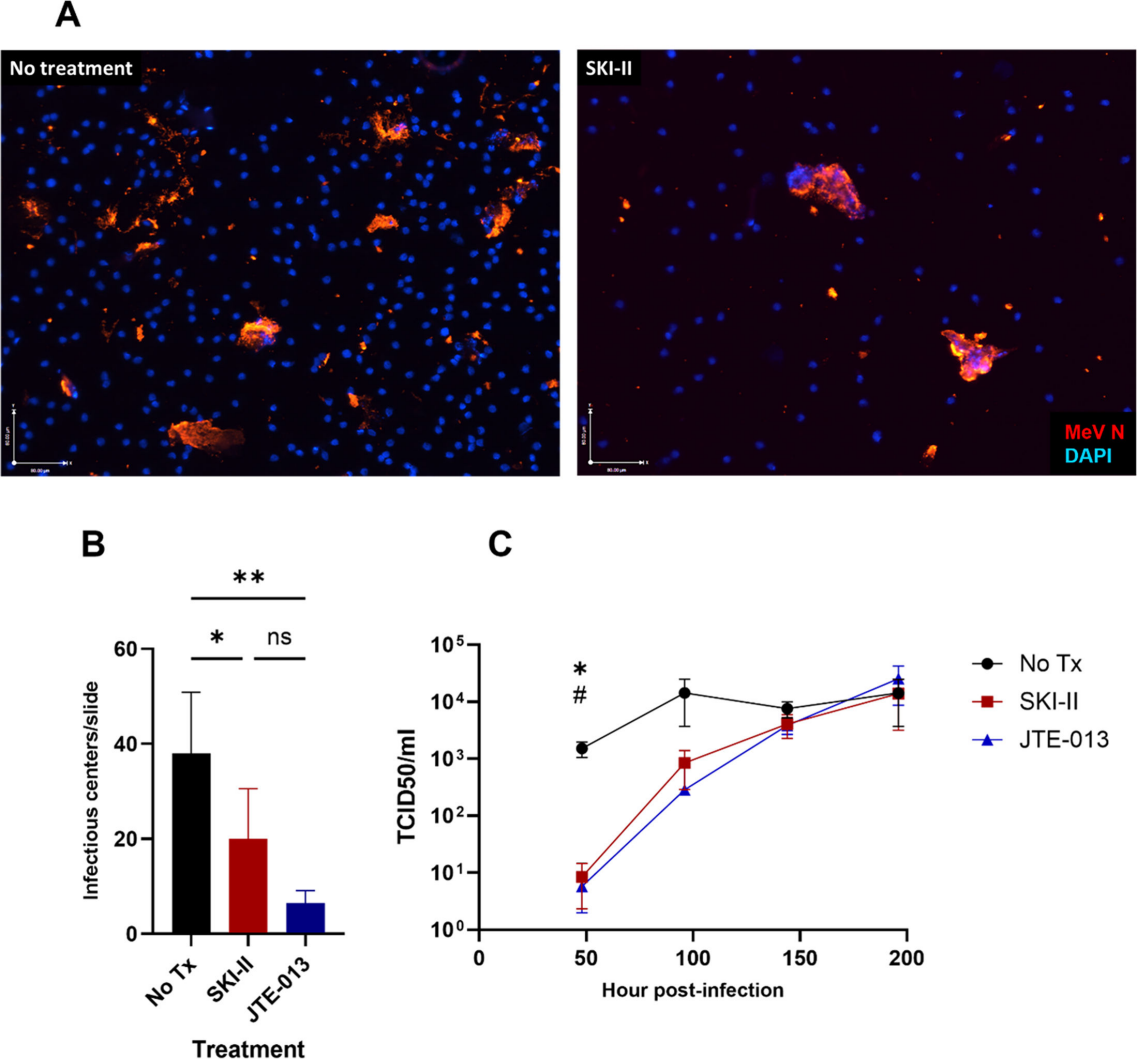
S1P signaling inhibition decreased shedding of MeV infectious centers from the epithelium. rhTECs were apically infected with WT MeV, and an inhibitor or vehicle was added to the basolateral media (MOI 1; *N* = 2–3 replicate wells per timepoint, from two donors). (A) Representative images of immunostained slides prepared by cytocentrifugation of cells present in the apical wash of infected rhTEC cultures. Images were taken at 10× magnification and show MeV-positive multinucleate infectious centers, along with uninfected shed cells. (B) Quantification of shed infectious centers at 192 h pi by microscopy (*SKI-II: *P* = 0.042; **JTE-013: *P* = 0.002). (C) MeV titers in shed cells from inhibitor-treated and untreated cultures were determined by co-cultivation of rhTECs on Vero-hSLAM cells (*SKI-II: *P* = 0.015; #JTE-013: *P* = 0.015). Significance was determined by one-way ANOVA (B) or two-way ANOVA (C) with Tukey’s multiple comparisons.

To examine the effects of S1P signal inhibition on MeV-infected epithelial cell shedding, we assessed supernatant fluids of WT MeV-infected rhTEC cultures treated with SKI-II and JTE-013. Apical washes were performed at 192 hpi, and cytocentrifugation was performed to observe infected cells shed into the apical chamber (Fig. 5A). SKI-II- and JTE-013-treated cultures had significantly fewer clusters of infected cells in the apical wash at 192 hpi compared with infected, untreated cultures (Fig. 5B). We also observed that the multi-cell clusters present in the inhibitor-treated cultures were generally larger than the infected multinucleate cells in the untreated cultures, possibly because the delayed release of the infected cells from the epithelium allowed for more lateral cell-to-cell spread within the epithelium (Fig. 5A).

Cell-associated virus within apically shed infected cells is the major source of virus produced by infected respiratory epithelial cultures (10, 11). We therefore quantified the amount of cell-associated virus present in the apical wash of treated and untreated rhTEC cultures. Shed epithelial cells in the apical wash were separated out by centrifugation and then co-cultivated with Vero-hSLAM cells. At 48 hpi, SKI-II- and JTE-013-treated cultures had produced significantly less infectious virus than untreated cultures (Fig. 5C). At later timepoints (96–192 hpi), amounts of virus produced by the apically shed cells of treated cultures increased to levels similar to untreated cultures. Overall, these data show that inhibiting cellular extrusion through S1P signaling results in decreased apical shedding of MeV-infected respiratory epithelial cells.

### Cell shedding begins prior to cluster formation during apical infection

**Fig 6:**
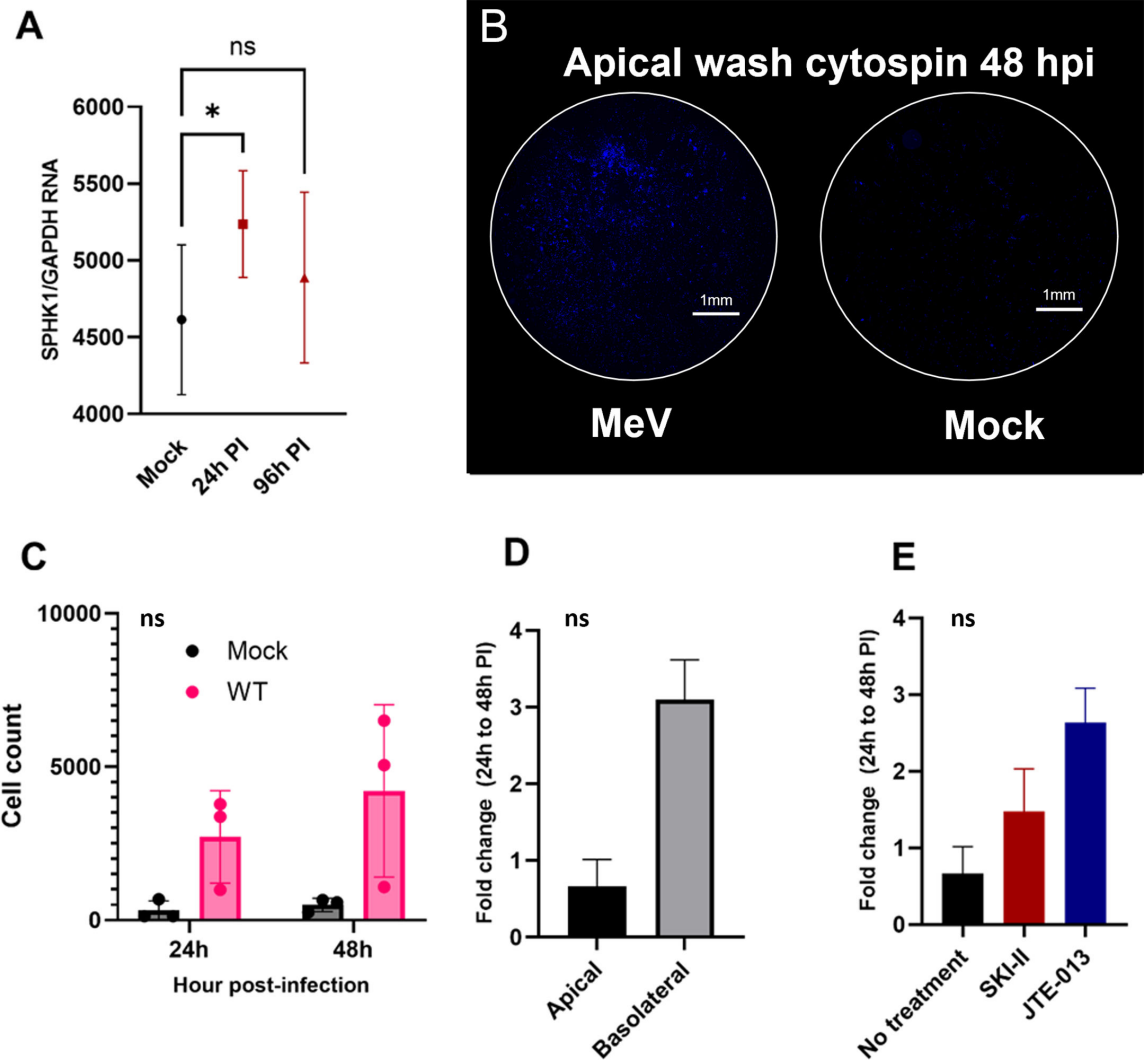
MeV-infected cell shedding begins within the first 48 h pi. (A) SPHK1 transcripts were quantified at 24 and 96 hpi in WT-infected rhTECs and compared to mock-infected cells (*P* = 0.05). (B) Representative images of cytospins prepared from apical washes of WT- and mock-infected rhTECs at 48 hpi. Nuclei are stained with DAPI. (C) Quantification of total cell numbers in apical wash of WT- and mock-infected cultures. ImageJ was used to quantify nuclei on cytospin preparations. (D) rhTECs were infected with WT MeV via the apical or basolateral route and then immunostained for MeV N protein (MOI 1, *n* = 3 replicate wells per donor, from three donors). MeV-positive cells were counted at 24 and 48 h pi, and fold change was calculated by dividing the 48 h count by the 24 h count. (E) Fold change experiments were repeated with the addition of inhibitors.

Previous research has shown that apical infection of polarized AEC cultures results in fewer detectable clusters of infected cells within the epithelium compared to infection via the basolateral route (10, 33). The shedding of cells after an apical infection might account for the absence of large clusters of infected cells in the epithelium. To investigate the kinetics of MeV-infected cell shedding, we first performed RT-qPCR to measure sphingosine kinase 1 (SPHK1) transcripts within the rhTEC epithelium during the first 96 h of apical infection with WT MeV. There was a transient, significant increase in SPHK1 transcripts in infected cultures at 24 hpi compared to mock-infected cultures (Fig. 6A), which decreased by 96 hpi. We also noted a trend showing an overall increase in shed cells present in the apical wash of MeV-infected cultures at 24 and 48 hpi compared to mock-infected cultures (Fig. 6B and C) suggesting that cell turnover in the epithelium of MeV-infected cultures may be higher than uninfected cultures.

We next used fluorescence microscopy to examine WT MeV-infected rhTECs at 24 and 48 hpi for changes in the presence of infected cells following apical or basolateral infection. After both apical and basolateral infection, occasional MeV-positive cells could be detected within the epithelium at 24 hpi. By 48 hpi, we observed a decrease in the number of individual WT MeV-positive cells in apically infected cultures compared to the numbers at 24 hpi (Fig. 6D; mean fold change = 0.67), whereas the number of infected cells increased in basolaterally infected cultures (Fig. 6D; mean fold change = 3.1). When apically infected cultures were treated with SKI-II or JTE-013, this loss was reversed (Fig. 6E; SKI-II mean fold change = 1.5; JTE-013 mean fold change = 2.6). These data, along with transient increases in SPHK1 at 24 hpi, suggest that S1P-driven cell shedding may be induced early during apical infection of the epithelium.

**Fig 7:**
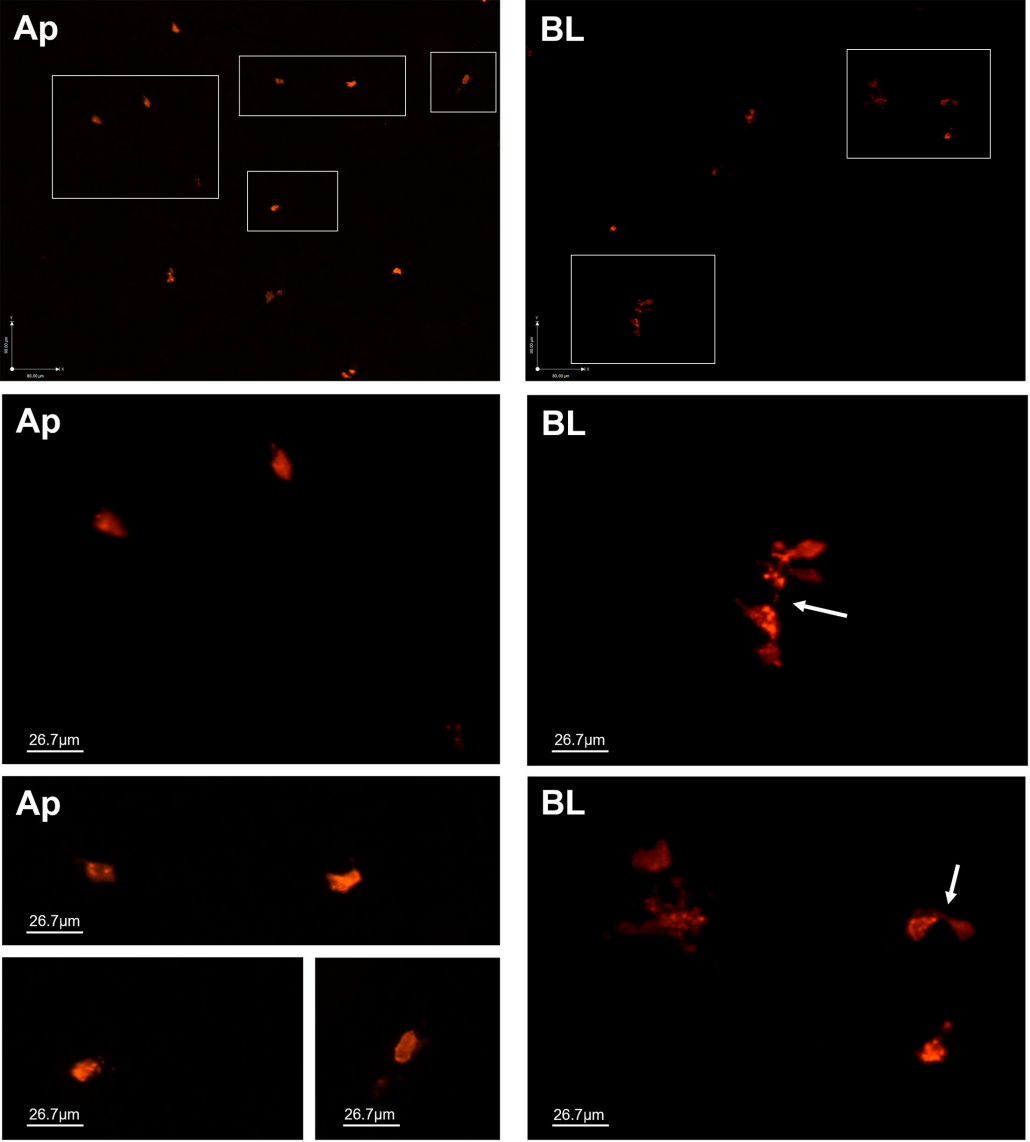
Morphology of MeV-positive cells may be different based on route of infection. rhTECs were infected with rGFP-LAMV via the apical or basolateral route, and epithelia were imaged at 48 h pi. Representative *en face* images show infected cell morphology after apical infection (Ap) and basolateral infection (BL). Cells within white boxes are magnified 3×. Cells in apically infected cultures tended to be solitary, while cells in basolaterally infected cultures formed small clusters with thin connections between cells (white arrow).

Interestingly, when we repeated these experiments using rGFP-LAMV to visualize cytoplasmic morphology, we noted differences by 48 hpi depending on the route of infection. In apically infected cultures, MeV-positive cells tended to be singular (Fig. 7). In basolaterally infected cultures, infected cells were frequently found in small clusters with thin, GFP-positive connections between them consistent with previously reported cytoplasmic streaming between neighboring MeV-infected cells (Fig. 7, white arrow) (27). GFP signal was not detectable before 24 hpi in both infection conditions. Based on these observations, we hypothesize that apical infection leads to loss of individually infected cells before significant cell-to-cell spread can occur, resulting in decreased frequency of clusters of infected cells compared to basolateral infection.

## DISCUSSION

In this study, we investigated the mechanisms driving shedding of MeV-infected respiratory epithelial cells using rhTEC cultures and samples from rhesus macaques and showed that SIP-mediated extrusion is the main mechanism for shedding of infected cells. Previous research has shown that MeV infection from both the apical and basolateral surface of polarized epithelial cells leads to shedding of viable, metabolically active clusters of infected cells that are capable of producing infectious viruses (10, 11). These infected cells leave the epithelium without evidence of cytopathology, such as loss of transepithelial resistance, and can remain viable for up to 48 h after losing contact with other cells in the epithelium.

We explored several pathways known to induce cell shedding in the context of other epithelial infections. Programmed cell death, whether pathogen-induced or as a host defense mechanism, can trigger exfoliation of infected cells (20–23). We confirmed that cell death did not precede infected cell shedding. There was no evidence of LDH release or consistent TUNEL staining in rhTECs infected with WT MeV or LAMV, or tracheal sections from LAMV-inoculated macaques. Furthermore, we found no evidence of pyroptosis or inflammasome activation in rhTECs or BAL samples from WT MeV-infected macaques. These data indicated that signals independent of apoptosis and pyroptosis drive the shedding of epithelial cells during MeV infection.

We, therefore, assessed the role of S1P-mediated cell shedding—a mechanism responsible for homeostatic removal of unwanted epithelial cells from the epithelium while maintaining barrier integrity—in the extrusion of MeV-infected cells. Inhibiting S1P production and blocking signaling through S1PR2 resulted in higher viral titers in the epithelium over time and initially lower titers in the apical wash of infected cultures. Examination of shed infected cells by microscopy showed that fewer clusters of infected cells were present in inhibited cultures, and that shed clusters tended to be larger in inhibited cultures than in untreated cultures. We hypothesize that S1P inhibition delays the release of infected cells, allowing further cell-to-cell spread within the epithelium before cells are extruded *en masse*. This may also explain why the amount of virus produced by the apically shed cells of treated cultures increased to levels similar to untreated cultures at later timepoints (96–192 hpi). It is possible that, although fewer clusters of infected cells were shed as a result of S1P signaling inhibition, dislodged clusters were larger and contained more virus. Mechanical disruption of clusters of infected cells contained within the epithelium may also occur during the washing process, freeing otherwise un-shed cells. Despite higher titers of virus being present in the epithelium in inhibited cultures, transepithelial resistance was maintained, indicating that increased viral loads did not cause greater damage to the epithelium.

Sphingolipids and their metabolites are key regulators of many cellular processes, including metabolism, growth, survival, and differentiation, and therefore have the potential to regulate replication of intracellular pathogens such as viruses (31, 34, 35). In response to proinflammatory cytokines and stress, S1P can be rapidly generated through the phosphorylation of sphingosine by SphK. The SphK/S1P pathway has anti-apoptotic, cell proliferation, and survival effects (31, 35). Interestingly, SphK and S1P regulate MeV replication in lymphocytic cell lines and primary peripheral blood mononuclear cells (PBMCs) (31, 32, 36). Infection of a human B-cell line with MeV resulted in a transient increase in S1P between 0.5 and 6 hpi (32). The expression and phosphorylation of SphK were increased in B95-8 marmoset B cells, although this increase was not noted in H358 epithelial-like cells, suggesting that SphK is differentially regulated depending on cell type (31). We demonstrated a similar transient increase in SphK transcripts at 24 hpi in our primary differentiated epithelial culture system, as well as increased SphK immunostaining in sections of LAMV-infected macaque tracheal tissue.

In primary PBMCs and B cell lines, inhibition of sphingolipid metabolism with SKI-II results in reduced replication of MeV, implying a pro-viral role for SphK and S1P. SKI-II treatment reduces the translational capacity of PBMCs, resulting in decreased production of MeV viral proteins and a 10-fold reduction in titers (32, 36). In addition, SphK and S1P bind TNF receptor-associated factor 2 (TRAF2) to activate TNF-α-induced NF-κB signaling (37). SKI-II treatment suppresses MeV-induced activation of NF-κB, which may have dual roles in both viral protein synthesis and anti-viral functions (31). In influenza virus infection, SphK-mediated activation of the NF-κB pathway, which promotes influenza viral RNA synthesis, has been described (34).

We did not observe a reduction in viral titers in either WT MeV- or LAMV-infected epithelial cultures treated with SKI-II. This may be due to differences in the innate responses of primary epithelial cells compared to those of lymphocytes. For example, LAMV replicates as well as—if not better than—WT MeV in rhTEC cultures, but LAMV viral particle production is restricted in PBMCs (38, 39). Interestingly, Vijayan et al. (28) demonstrated that overexpression of intracellular SK1 enhances MeV replication, while exogenously added S1P, which signals through S1PR2, did not affect MeV replication. In our experiments, viral titers were generally higher in the rhTECs treated with the S1PR2 inhibitor JTE-013 compared with SKI-II-treated cultures. It is possible that SKI-II inhibition of intracellular S1P levels had some effect on MeV replication, while JTE-013 did not impact overall MeV replication.

This study did not explore the triggers of S1P signaling that occur after infection. Pattern recognition receptors (PRRs) sensing the MeV genome or viral proteins may induce activation of SphK. For example, stimulation of TLR4 by LPS activates SphK (40). Influenza virus increases the expression and phosphorylation of Sphk1 in human lung epithelial A549 cells (34). Proinflammatory cytokines also stimulate sphingolipid metabolism (41). Further work will be needed to identify the signals driving S1P-pathway activation within epithelial cells after MeV infection.

Epithelial cell extrusion may also provide an opportunity for MeV to gain access to nectin-4 from the apical surface of the epithelium, as normally obscured junctional proteins are exposed. This is used by *Listeria monocytogenes*, which invade intestinal epithelial cells through basolateral proteins only exposed during the extrusion process (18, 42). Microscopy performed on rhTEC cultures infected from the apical or basolateral surface with rGFP-LAMV revealed distinct cell morphologies, with cells in apically infected cultures appearing singularly and cells in basolaterally infected cultures appearing more frequently in clusters. It is possible that individually infected cells in apical cultures are cells already in the process of extruding, and therefore susceptible to MeV infection through exposed nectin-4. By contrast, cells infected through the basolateral route may still be embedded in the epithelium, facilitating cell-to-cell spread of MeV as previously described (27, 43). A caveat to this explanation is that LAMV receptor tropism includes CD46, which is expressed on all nucleated cells and therefore might not accurately represent WT MeV interactions with epithelial cells. GFP production by rGFP-LAMV stains the entire cytoplasm of infected cells, making it easier to detect subtle morphologic changes by microscopy compared with single-protein staining. When immunofluorescent imaging was repeated with WT MeV and staining for N protein, we could not confidently determine cell morphology. Other explanations for these morphological variances include differences in the cell type initially infected. While MeV is known to primarily infect ciliated epithelial cells, basal cells express nectin-4 (11, 33). It is possible that basolateral infection may occasionally lead to infection of basal cells that are located at the base of the polarized, pseudostratified epithelium, possibly changing viral kinetics within the epithelium.

Ultimately, it is not clear whether MeV-infected cell shedding is a host defense response, an advantageous strategy for viral spread, or a combination of both. Exfoliation of epithelial cells has been described as a mechanism of pathogen clearance in bacterial and parasite worm infections within the gastrointestinal tract (14, 44, 45). Inhibiting shedding led to increased viral titers in the epithelium with no appreciable damage to the integrity of the epithelium, but it is unclear the ultimate effect this had on transmission of virus. Inhibiting cell shedding decreased viral titers early after infection, but eventually, the titers of inhibited cultures equaled those of uninhibited cultures. We suspect this is due to the generally higher titers of virus within the rhTEC culture system at later timepoints. As previously postulated, the shedding of infected cells may allow for efficient viral transmission both between and within hosts (10, 11).

Our study has several limitations. AEC cultures have intact innate immune responses but lack the immune cells normally present in airways. Using an AEC model of SARS-CoV-2 infection, Barnett et al. found that bi-directional interactions between infected respiratory epithelium and leukocytes added to the culture system were necessary for production of IL-1β and IL-6 (46). UPEC-induced bladder epithelial cell shedding is triggered after cells take up granules released by local mast cells recruited by epithelial inflammatory signals (21). As our cultures only contain differentiated epithelial cells (47), it is possible that we are missing important components of MeV behavior and host response in the respiratory epithelium. However, our data from macaque samples support the *in vitro* work we performed, suggesting that the rhTEC model system is robust in this context. Because we regularly work in rhesus macaque models, we opted to perform our experiments in AEC cultures derived from macaques. It is possible that mechanistic differences exist between macaques and humans that were not captured in our culture system. Further work can be performed in human AECs to confirm the findings presented here. Finally, we used rhTEC cultures in passages one and two for experiments. Kaufman et al. demonstrated that primary differentiated airway epithelial cultures that were passaged twice using BronchiaLife and USG media had a loss of epithelial morphology and basolateral-specific MeV infection (48). We used PneumaCult media and did not see altered morphology at passage two (Fig. S1). Therefore, we believe that our rhTEC cultures accurately reflect the biology of the tissue we are modeling.

In conclusion, both WT MeV and LAMV induce shedding of infected respiratory epithelial cells through S1P signaling, and inhibition of S1P production or signal reception results in altered viral dynamics within the respiratory epithelium. SKI-II and other sphingolipid inhibitors have been proposed as measles antiviral treatments based on work performed in human lymphocytes (32, 36). Our work suggests that S1P signal inhibition in the airways may be more complicated. Future work might include sphingolipid-inhibitor treatment of MeV-infected macaques to assess the effects on overall spread of virus within the host and transmission potential between hosts.

## MATERIALS AND METHODS

### Cell lines and viruses

All MeV stocks were grown and assayed by plaque formation in Vero cells stably expressing human SLAM (Vero/hSLAM) (49). Vero/hSLAM cells were grown in Dulbecco’s modified Eagle’s medium (DMEM; Gibco, Life Technologies Corporation, Grand Island, NY, USA) supplemented with 10% fetal bovine serum (FBS; Gibco) and 1% penicillin/streptomycin (pen/strep; Gibco). DMEM supplemented with 1% FBS (1% DMEM; inoculation media) was used during MeV infection of Vero/hSLAM cells. The following strains of MeV were used: Bilthoven WT MeV (genotype C2; a gift from Albert Osterhaus, Erasmus University, Rotterdam, Netherlands); Edmonston-Zagreb strain of LAMV (Serum Institute of India); recombinant GFP-expressing EZ (rGFP-LAMV) strain of LAMV. rGFP-LAMV contains an additional transcriptional unit expressing GFP after the P gene as previously described (50). Influenza A virus A/Baltimore/R0243/2018 H3N2 was used as a positive control for inflammasome activation in AECs (gift from Andrew Pekosz, Johns Hopkins Bloomberg School of Public Health, Baltimore, MD).

### Samples from MeV-infected macaques

Samples from previous studies on WT MeV or LAMV-infected rhesus macaques were used. All procedures were approved by the Johns Hopkins University Institutional Animal Care and Use Committee and conducted in accordance with guidelines in the Animal Welfare Regulations and the Guide for the Care and Use of Laboratory Animals within a fully AAALAC-accredited facility. In brief, macaques were intratracheally infected with 10^4^ PFU of WT MeV or LAMV. LAMV-inoculated animals were necropsied at 11 dpi, and tissues were fixed in 10% formalin and embedded in paraffin prior to slide preparation. For WT MeV-infected macaques (*n* = 4), BAL samples were obtained through the modified mini-BAL method using phosphate-buffered saline (51). BAL supernatants were aliquoted and stored at −80C prior to cytokine analysis.

### Rhesus macaque rhTEC cultures

Tracheal epithelial cells were collected from rhesus macaque (*Macaca mulatta*) tracheas obtained at necropsy (10, 52). In brief, tracheas were excised from below the larynx to the bronchial bifurcation and placed in 10× PBS for transportation to the laboratory. Excess tissue was removed, and the trachea was cut lengthwise. The entire trachea was placed into 0.2% pronase (Sigma Aldrich, St. Louis, MO) in Ham’s F-12 with 100 U/mL penicillin and 100 ug/mL streptomycin (Life Technologies) at 4°C for 12–18 h, followed by successive washes with Ham’s F-12 supplemented with 10% FBS. At each wash, tracheal tissue was vigorously inverted to dislodge epithelial cells. Dislodged cells were combined and centrifuged at 300 G for 10 min, and the cell pellet was suspended in a 0.5 mg/mL DNase solution (Sigma) for 10 min at room temperature. Cells were then centrifuged and resuspended in PneumaCult-Ex Plus Medium (Stemcell Technologies, Cambridge, MA) supplemented with 1% pen/strep and 250 ng/mL amphotericin B (Gibco) and plated on a plastic culture dish, incubating at 37°C, 5% CO_2_ for 4 h to allow fibroblasts to adhere. Unadhered tracheal epithelial cells were collected and expanded on rat-tail I collagen-coated T75 flasks (50 μg/mL; Gibco) until 80% confluent, at which point cells were collected with 2× trypsin (Gibco) for differentiation or freezing in liquid nitrogen. Cells at passages 1–2 were used for experiments.

TECs were cultured on collagen-coated 6.5 mm or 12 mm Transwell 0.4 μm-pore polyester membrane inserts (Corning Costar, Corning, NY). Cells were plated at a density of 10^5^ cells/cm^2^ and grown in PneumaCult-Ex Plus Medium supplemented with 1% pen/strep and amphotericin B until confluent, at which point media was lifted from the apical chamber and cells were maintained at the air-liquid interface (ALI) for 3–4 weeks until fully differentiated. PneumaCult-ALI Medium supplemented with 1% pen/strep and 250 ng/mL amphotericin B was added to the basolateral chamber during the ALI phase and replaced every 2 days. To remove excess mucus, 1× PBS was added to the apical chamber every 2 weeks, and cultures were incubated at 37°C for 15–30 min before aspirating. Differentiation was confirmed by transepithelial electrical resistance greater than 800 Ω-cm^2^, and observation of cilia movement. Appropriate epithelial differentiation and morphology were further confirmed by immunostaining for zonula occludens-1 (ZO-1) protein and hematoxylin and eosin staining of formalin-fixed, paraffin-embedded (FFPE) rhTEC cross-sections (Fig. S1).

### MeV infection of rhTEC cultures

At least two different macaque donors and three replicate wells were used for each experiment. Prior to infection, mucus was washed from the culture surface as described above. For apical infection, virus in inoculation media was added at an MOI of 1 to the apical chamber. For basolateral infection, inserts were inverted, and inoculum was added at an MOI of 1 to the basal surface of the membrane. Cultures were inoculated for 2 h at 37°C, 5% CO_2_. Following infection, apical or basal surfaces were washed once with 1× PBS, and basolateral media was replaced.

Throughout the course of infection, sample collection was performed. To perform an apical wash, 200–500 μL 1% DMEM or PBS was added to the apical chamber and incubated for 30 min. Washes were centrifuged at 600 G for 10 min to isolate cells. To collect adherent epithelial cells for PCR or TCID_50_ assays, 2× trypsin was added to apical and basolateral chambers, and cultures were incubated for 15 min before removing disassociated cells. Basolateral media and apical supernatant fluids were frozen at −80°C.

### S1P signaling inhibition

The sphingosine kinase 1/2 inhibitor SKI-II (Abcam, Cambridge, MA) and sphingosine-1-phosphate receptor antagonist JTE-013 (Sigma) were used to inhibit S1P signaling in rhTEC cultures. Inhibitors were prepared in DMSO and added to the basolateral media every 48 h at media change for a total of 10 µM per 500 µL. A corresponding 1 µL volume of DMSO was added to control wells.

### Immunofluorescent staining

FFPE tissues from rhesus macaques intratracheally inoculated with LAMV or rhTEC cultures were used for immunofluorescent staining assays. FFPE tissues were baked at 58°C and rehydrated, followed by antigen retrieval with 1 mg/mL proteinase K (Thermo Fisher Scientific Baltics UAB, Lithuania) for 20 min. Cells collected in the apical wash were incubated for 15 min in a 1:1 solution of 0.1% dithiothreitol (DTT) (Sigma) in PBS for mucus dissolution. Cells were resuspended to ~4 × 10^4^ cells/100 µL and cytocentrifuged (700 G × 4 min) onto glass slides, then fixed for 10 min with 4% paraformaldehyde prior to staining. Adherent epithelial cells were fixed for 10 min with 4% paraformaldehyde, and membranes were cut from inserts so staining could be performed in 48 well plates. Cells or tissues were permeabilized with 0.2% Triton X-100 (Sigma) for 20 min, then blocked with 2% normal goat serum/0.5% BSA for 1 h, followed by primary antibody for 1 h and secondary antibody for 1 h. The following antibodies were used: anti-MeV N protein (1:200; MAB8906, clone 83KKII, Millipore, Burlington, MA), anti-SPHK1 (1:200; PA514068; Life Technologies), ZO-1 (1:200; ZO1-1A12; Invitrogen), Alexa Fluor 594 (1:1000; A-11005; Life Technologies), and Alexa Fluor 488 (1:1,000; A-11008; Life Technologies). Cells were incubated with DAPI (Life Technologies) for 15 min prior to mounting with ProLong Gold Antifade (Life Technologies). TUNEL staining was performed on adherent epithelial cells and macaque tissues using the Click-iT-Plus TUNEL Assay for *in situ* Apoptosis Detection kit (Life Technologies) according to the manufacturer’s instructions. Imaging was performed using a Zeiss AxioImager M2 fluorescent microscope and Leica Thunder Imager Live Cell fluorescent microscope. Images were processed and analyzed with Image J software.

### Lactate dehydrogenase quantification

The CyQUANT LDH Cytotoxicity Assay (Life Technologies) was used according to the manufacturer’s instructions to quantify LDH release from rhTEC cultures. Basolateral fluid from mock and infected cultures was collected every 48 h. For positive control, rhTEC wells were incubated with Triton-X 100 for 24 h to induce maximal cell membrane disruption and LDH release. Percent cytotoxicity was calculated according to the manufacturer’s instructions.

### RT-qPCR

MeV N gene RNA in rhTEC cells was quantified by RT-qPCR as previously described (53, 54). RNA was extracted with Qiagen RNeasy Mini or Micro kits (Qiagen, Germantown, MD) according to the manufacturer’s instructions. The MeV N gene was amplified using a TaqMan RNA-to-CT one-step RT-PCR kit (Applied Biosystems, Waltham, MA) with MeV N specific primers (MVN Forward: 5′-GGGTACCATCCTAGCCCAAATT-3′; MVN Reverse: 5′-CGAATCAGCTGCCGTGTCT-3′) and probe (5′-CTCGCAAAGGCGGTTACGGCC). RT-qPCR was performed with an ABI Prism 7700 Sequence Detection System (Life Technologies). Copy number was determined by the construction of a standard curve from 1 to 10^8^ copies of RNA synthesized by *in vitro* transcription of a plasmid encoding the Edmonston MeV N gene. The housekeeping gene GAPDH was used for control (TaqMan GAPDH Control Reagents kit, Applied Biosystems). Data were normalized to GAPDH and expressed as [(copies of MeV N RNA)/(copies of GAPDH RNA)]  × 10E10.

For SPHK1 quantification, the following primers were used: Forward GTGCTGGTGCTGAA & Reverse GCATCAGCGTGAAGGAGATT. cDNA was synthesized using SuperScript III Reverse Transcriptase (Invitrogen), and qPCR was performed using SYBR Green Master Mix (Bio-Rad, Hercules, CA). SPKH1 levels were expressed as (5000/[SPKH1 CT/GAPDH CT]).

### Infectious virus assay

Infectious virus in shed and adherent epithelial cells from rhTEC cultures were quantified by overlay onto vero/hSLAM cells as previously described (54). Vero/hSLAM cells were grown to confluence on 48 well plates. Epithelial cells were suspended in 1% DMEM and serially diluted before being added to the vero/hSLAM monolayer in triplicate. Plates were incubated for 5 days at 37°C, 5% CO_2_. After the 5th day, wells were assessed for the presence of MeV cytopathic effect, and TCID_50_ was determined using the methods of Reed and Muench (55).

### Statistics

Statistical analysis was performed using GraphPad Prism 10. Data are presented as means ± standard errors of at least three independent experiments or replicate wells. A *P* value of < 0.05 was considered significant. Statistical analyses are specified in each figure legend.

## Data Availability

Data not included in the article will be made available by the authors upon request.

## SUPPLEMENTAL MATERIAL

Figure S1 jvi.01880-24-s0001.tif Morphology of fully differentiated rhTEC cultures.

Supplemental legend jvi.01880-24-s0002.docx Legend for Fig. S1.

ASM does not own the copyrights to Supplemental Material that may be linked to, or accessed through, an article. The authors have granted ASM a non-exclusive, world-wide license to publish the Supplemental Material files. Please contact the corresponding author directly for reuse.
