# Supplementary figures and images for: Sphingosine-1-phosphate signaling mediates shedding of measles virus-infected respiratory epithelial cells
Source: J Virol. 2025 Mar 27;99(4):e01880-24. doi: 10.1128/jvi.01880-24 (PMC11998495; doi:10.1128/jvi.01880-24)

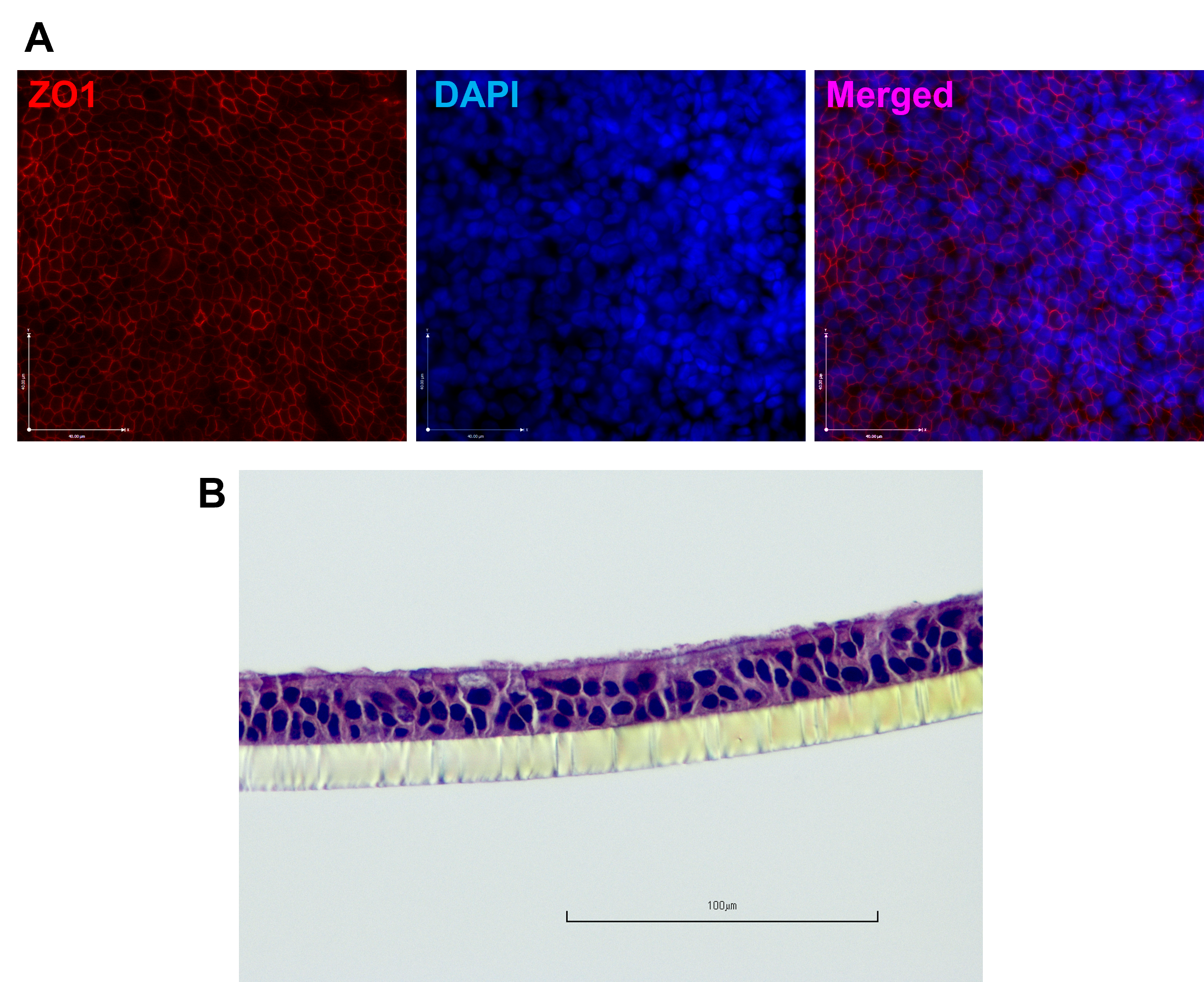

Supplement: Figure S1 — Morphology of fully differentiated rhTEC cultures. [file jvi.01880-24-s0001.tif]
