# Supplementary material for: Sphingosine-1-phosphate signaling mediates shedding of measles virus-infected respiratory epithelial cells
Source: J Virol. 2025 Mar 27;99(4):e01880-24. doi: 10.1128/jvi.01880-24 (PMC11998495; doi:10.1128/jvi.01880-24)
Supplement: Supplemental legend — Legend for Fig. S1. [file jvi.01880-24-s0002.docx]

Figure S1. Morphology of fully differentiated rhTEC cultures. (A) ZO-1 immunostaining was performed to stain tight junctions between cells in the epithelium. (B) Representative cross section of rhTECs on a Transwell insert, stained with hematoxylin and eosin. Cells form a pseudostratified, ciliated, columnar epithelium.
